# Relationship between protein biomarkers of chemotherapy response and microsatellite status, tumor mutational burden and PD‐L1 expression in cancer patients

**DOI:** 10.1002/ijc.32661

**Published:** 2019-10-01

**Authors:** Mina Nikanjam, David Arguello, Zoran Gatalica, Jeff Swensen, Donald A. Barkauskas, Razelle Kurzrock

**Affiliations:** ^1^ Center for Personalized Cancer Therapy, Division of Hematology and Oncology University of California San Diego Moores Cancer Center San Diego CA; ^2^ Caris Life Sciences, Inc Phoenix AZ; ^3^ Department of Preventive Medicine, Biostatistics Division, Keck School of Medicine University of Southern California Los Angeles CA

**Keywords:** immunotherapy, cytotoxic chemotherapy, PD‐L1, MSI, TMB

## Abstract

Chemotherapy and checkpoint inhibitor immunotherapies are increasingly used in combinations. We determined associations between the presence of anti‐PD‐1/PD‐L1 therapeutic biomarkers and protein markers of potential chemotherapy response. Data were extracted from a clinical‐grade testing database (Caris Life Sciences; February 2015 through November 2017): immunotherapy response markers (microsatellite instability‐high [MSI‐H], tumor mutational burden‐high [TMB‐H], and PD‐L1 protein expression) and protein chemotherapy response markers (excision repair complementation group 1 [ERCC1], topoisomerase 1 [TOPO1], topoisomerase 2 [TOP2A], thymidylate synthase [TS], tubulin beta 3 [TUBB3], ribonucleotide reductase regulatory subunit M1 [RRM1] and O‐6‐methyl guanine DNA methyltransferase [MGMT]). Relationships were determined by the Mantel‐Haenszel chi‐squared test or Fischer's exact tests. Overall, 28,034 patients representing a total of 40 tumor types were assessed. MSI‐H was found in 3.3% of patients (73% were also TMB‐H), TMB‐H, 8.4% (28.3% were also MSI‐H) and PD‐L1 expression in 11.0% of patients (5.1% were also MSI‐H; 16.4% were also TMB‐H). Based on concurrent biomarker expression, combinations of immunotherapy with platinum (ERCC1 negativity) or with doxorubicin, epirubicin or etoposide (TOP2A positivity) have a higher probability of response, whereas combinations with irinotecan or topotecan (TOPO1 positivity), with gemcitabine (RRM1 negativity), and fluorouracil, pemetrexed or capecitabine (TS negativity) may be of less benefit. The potential for immunotherapy and taxane (TUBB3 negativity) combinations is present for MSI‐H but not TMB‐H or PD‐L1‐expressing tumors; for temozolomide and dacarbazine (MGMT negative), PD‐L1 is frequently coexpressed, but MSI‐H and TMB‐H are not associated. Protein markers of potential chemotherapy response along with next‐generation sequencing for immunotherapy response markers can help support rational combinations as part of an individualized, precision oncology approach.

## Abstract 2

**What's new?**

With the emerging success of immunotherapy of cancers, combinations with conventional chemotherapies are increasingly being tested in clinical trials. Here the authors examined concurrent biomarker expression of checkpoint (PD‐1/PD‐L1) blockade immunotherapy and various cytotoxic chemotherapies to determine which chemotherapeutic agents will best synergize with immunotherapy. They predict that combining platinum or doxorubicin, epirubicin, or etoposide treatments with PD‐1/PD‐L1 inhibitors would have a higher probability of response than other treatments, supporting a rational combination strategy in a possibly individualized treatment approach.

**Abbreviations**
ERCC1: excision repair complementation group 1
FDA: Food and Drug Administration
IHC: immunohistochemical analysis
M‐H: Mantel‐Haenszel
MGMT: O‐6‐methyl guanine DNA methyltransferase
MSI‐H: microsatellite instability‐high
NGS: next‐generation sequencing
RRM1: ribonucleotide reductase regulatory subunit M1
TMB‐H: tumor mutational burden‐high
TOP2A: topoisomerase 2
TOPO1: topoisomerase 1
TS: thymidylate synthase
TUBB3: tubulin beta 3

## Introduction

Combinations of immunotherapy and cytotoxic chemotherapy are increasingly being used and tested in clinical trials.^1^, ^2^, ^3^ Chemotherapy has the potential to enhance antitumor immune responses^4^ by several mechanisms including activation of immune effectors such as monocytic‐derived dendritic cells^5^ and sensitizing tumor cells to lysis.^6^, ^7^ However, preclinical studies have shown that chemotherapy can also deplete immunosuppressive cells, including myeloid‐derived suppressor cells^8^ and T‐regulatory cells.^9^, ^10^ It is unclear which cytotoxic chemotherapeutic agents will synergize best with immunotherapy. However, several biomarkers have been associated with responses to anti‐PD‐1/PD‐L1 checkpoint inhibitors: microsatellite instability high status (MSI‐H),^11^ high tumor mutational burden (TMB‐H),^12^, ^13^ programmed death‐ligand 1 (PD‐L1) amplification and increased expression of PD‐L1 on immunohisochemistry.^14^, ^15^, ^16^, ^17^, ^18^

Protein markers may aid in predicting response or resistance to specific cytotoxic chemotherapeutic agents (Supporting Information Table S1). Elevated topoisomerase 2 (TOP2A) expression has been linked to doxorubicin response in soft tissue sarcomas,^19^ whereas increased topoisomerase 1 (TOPO1) expression has been associated with response to irinotecan in colorectal cancer.^20^ Expression of TOP2A can also predict responses to etoposide and other anthracyclines.^21^ High thymidylate synthase (TS) was associated with decreased response to capecitabine in metastatic breast cancer,^22^ whereas low TS was associated with better response to 5‐fluorouracil in colorectal cancer^23^ and longer progression‐free survival with pemetrexed in nonsmall cell lung cancer.^24^, ^25^ Tubulin beta 3 (TUBB3) expression has been linked to resistance to taxanes in ovarian cancer and lower survival in prostate cancer.^26^, ^27^, ^28^ Expression of excision repair complementation group 1 (ERCC1) negativity predicts improved response in bladder cancer and longer survival in ovarian and gastric cancers in with the use of platinum agents.^29^, ^30^ O‐6‐methyl guanine DNA methyltransferase (MGMT) deficiency may predict response to dacarbazine in melanoma^31^ and temozolomide (glioblastoma and neuroendocrine tumors).^32^, ^33^ Ribonucleotide reductase regulatory subunit M1 (RRM1) negativity may predict response to gemcitabine in nonsmall cell lung cancer.^34^

The aim of the current study was to determine associations between protein expression markers of response to chemotherapy and immunotherapy response markers (MSI‐H, TMB‐H and PD‐L1 expression) in order to determine which immunotherapy and chemotherapy combinations could be more likely benefit various patient populations.

## Materials and Methods

### Patient population

Cases submitted to Caris Life Sciences (http://www.carislifesciences.com), a Clinical Laboratory Improvement Amendments‐certified laboratory, between February 2015 and November 2017 that had results for MSI status, TMB and immunohistochemical (IHC) analysis (PD‐L1, ERCC1, TOPO1, TOP2A, TS, TUBB3, RRM1 and/or MGMT) were analyzed. Tissue diagnoses were based on pathology reports from requesting physicians and were verified by a Caris laboratory‐based pathologist. Formalin‐fixed paraffin‐embedded tissues were processed as previously described.^35^ Patient identity protection was maintained throughout the study and the information reflected a deidentified database, so the study was considered exempt and institutional review board approval was waived.

### Techniques for evaluating markers

A variety of technologies were used to evaluate markers and are summarized in Supporting Information Table S2.

#### *Next‐generation sequencing*

MSI status and TMB were determined using next‐generation sequencing (NGS) analysis. NGS was performed on genomic DNA isolated from formalin‐fixed paraffin‐embedded tissue using a NextSeq platform (Illumina Inc., San Diego, CA). An Agilent custom‐designed SureSelectXT assay (Agilent Technologies, Santa Clara, CA) then was utilized to enrich the 592 whole‐gene targets that comprised the NGS panel (592 genes). All reported variants were detected with greater than 99% confidence, based on the frequency of the mutation present and the amplicon coverage. The average depth of coverage for this assay is ×500 with an analytic sensitivity of 5% variant frequency. To calculate TMB, the number of somatic nonsilent protein‐coding mutations with exclusion of copy number gene alterations and structural rearrangements were determined.^36^ TMB‐H was defined as greater than or equal to 17 mutations per megabase (Muts/Mb), TMB‐intermediate was 6–16 Muts/Mb, and TMB‐low <6 Muts/Mb.

MSI instability by NGS microsatellite loci in the targeted genes of the panel was first identified using the multiobjective immune system algorithm (MISA; 8,921 locations identified). Subsequent analyses excluding sex chromosome loci, microsatellite loci in regions that typically have lower coverage depth relative to other genomic regions and microsatellites with repeat unit lengths greater than five nucleotides led to 7,317 target microsatellite loci. After DNA was sequenced by NGS, the 7,317 target microsatellite loci were examined and compared with the reference genome hg19 from the University of California Santa Cruz Genome Browser database. The number of microsatellite loci that were altered by somatic insertion or deletion was counted for each patient sample and only insertions or deletions that increased or decreased the number of repeats were considered. A locus was not counted more than once even in the setting of multiple lengths of insertions or deletions. Thresholds were calibrated based on a comparison of total number of altered loci per patient to MSI‐fragment analysis results.^37^

#### *Immunohistochemical analysis*

IHC was performed on the tumor samples using commercially available detection kits and autostainers (BenchmarkXT, Ventana Medical Systems Inc., Santa Clara, CA and Autostainer Link 48, Dako). Primary antibodies used for protein detection were: ERCC1 (8F1) from Abcam (Cambridge, UK), TOPO1 (1D6) and TOP2A (3F6) from Leica Microsystems (Buffalo Grove, IL), MGMT (MT21.2) from Invitrogen (Carlsbad, CA), RRM1 (polyclonal) from Proteintech Group (Rosemont, IL), TS (TS106) from Dako), TUBB3 (PRB‐435P) from BioLegend) and PD‐L1 (SP142) from Ventana. The laboratory used staining protocols by Ventana Medical Systems, Inc. or the Dako (Carpinteria, CA) automated staining systems. Appropriate positive and negative control specimens were included for all the antibodies tested. Scoring for all slides was performed manually by board‐certified pathologists with results reported as a percentage of tumor cells that stained positive and intensity of staining (0, 1+, 2+ and 3+).

#### *Statistics*

All statistical analysis was verified by our biostatistician (DAB). Associations between MSI status, PD‐L1 expression or TMB status and protein markers (ERCC1, TOPO1, TOP2A, TS, TUBB3, RRM1 and MGMT) were analyzed with the Mantel‐Haenszel (M‐H) chi‐square test using tumor type as stratification. The association between the protein markers and the presence of any marker predicting response to immunotherapy (MSI‐H, TMB‐H or PD‐L1 expression) was also determined. The Breslow‐Day test was used to determine if the odds ratios (OR) for different tumor types were similar such that they could be combined in the analysis. If the Breslow‐Day test was not significant (*p* ≥ 0.05), then the M‐H statistic and adjusted OR were used to describe the data. If the Breslow‐Day test was significant (*p* < 0.05) then Fisher's exact test for each tumor type were used to determine significant relationships and the relationships were described by ORs in each tumor type separately. *p* Values less than or equal to 0.05 were considered significant.

### *Data availability*

The data that support the findings of this study are available from the corresponding author upon reasonable request.

## Results

Data were available for 28,034 patients with MSI status and classified by 40 cancer types (Table 1; Supporting Information Tables S3 and S4 ). Overall, MSI‐H was found in 3.3% of patients; TMB‐H, in 8.4% and PD‐L1 expression in 11.0% of patients. TMB‐H was found in 2,340 patients; of these, 662 (28.3%) were also MSI‐H and 24.9% of those tested expressed PD‐L1. TMB‐intermediate was found in 7,990 patients; of these, 1.6% (125) were MSI‐H. MSI‐H was found in 911 patients with a TMB result; of these 73% (662) were TMB‐H (125 patients had TMB‐intermediate and 124 patients, TMB‐low) and 15.4% of tested patients expressed PD‐L1. Of the PD‐L1 expressing tumors that were tested for MSI, 5.1% were MSI‐H; of the PD‐L1 expressing tumors that were tested for TMB, 16.4% were TMB‐H.

**Table 1:** Microsatellite status, tumor mutational burden and protein expression in 28,034 patients^1^

	Positive (%)	Low or negative (%)	Number of patients tested	Comment
MSI‐H	**3**.**3%**	96.7%	28,034	MSI‐H is one marker for checkpoint inhibitor immunotherapy response. Therefore, 3.3% of patients tested for MSI‐H status may benefit from checkpoint inhibitors.^11^
TMB‐H	**8**.**4%**	91.6%	27,847	TMB‐H is one marker of immunotherapy response.^12^
PD‐L1	**11**.**0%**	89.0%	22,114	PD‐L1 expression is a marker for immunotherapy response.^14^
ERCC1	20.9%	**79**.**1%**	21,802	ERCC1 negative correlates with platinum response^29^, ^30^; 79% of patients tested are ERCC negative/low.
MGMT	55.4%	**44**.**6%**	5,200	MGMT negative correlates with dacarbazine/temozolomide response^31^, ^32^, ^33^; 45% of patients tested have MGMT negative/low
RRM1	19.9%	**80**.**1%**	17,205	RRM1 negative correlates with gemcitabine response^34^; 80% of patients tested have RRM1 negative/low.
TOP2A	**75**.**8%**	24.2%	12,907	TOP2A positive correlates with doxorubicin,^19^ etoposide, epirubicin^21^ response; 76% of patients have TOPO2A high
TOPO1	**58**.**7%**	41.3%	22,211	TOPO1 positive correlates with irinotecan and topotecan response^20^; 59% of patients have TOPO1 positive disease
TS	34.0%	**66**.**0%**	20,491	TS negative correlates with fluorouracil/pemetrexed/capecitabine response^22^, ^23^, ^24^, ^25^; 66% of patients tested have TS negative/low.
TUBB3	56.8%	**43**.**2%**	19,863	TUBB3 positive correlates with taxane resistance^26^, ^27^, ^28^; 43% of patients tested have TUBB3 negative/low

**Figure 1:**
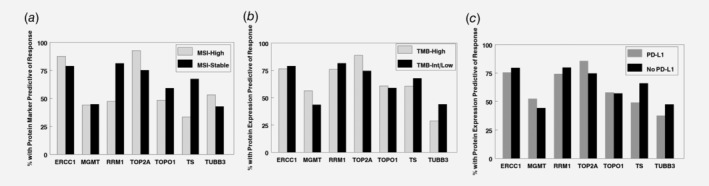
Protein markers predictive of response to chemotherapy compared with immunotherapy response makers. (*a*) MSI‐H (predictive of checkpoint inhibitor response) versus MSI‐stable. Eighty‐eight percent of MSI‐H also have ERCC1 negativity (predictive of platinum response); 44% of MSI‐H also have MGMT negativity (predictive of dacarbazine and temozolomide response); 47% of MSI‐H also have RRM1 negative (predictive of gemcitabine response); 93% of MSI‐H patients have TOP2A positivity (predictive of with doxorubicin, epirubicin, etoposide response), 48% of MSI‐H patients have TOPO1 positivity (predictive of irinotecan or topotecan response), 33% of MSI‐H patients have TS negativity (predictive of fluorouracil/pemetrexed/capecitabine response) and 53% of MSI‐H patients have TUBB3 negativity (predictive of taxane response). (*b*) TMB‐H (predictive of checkpoint inhibitor response) versus TMB‐intermediate/‐low. Seventy‐six percentage of TMB‐H also have ERCC1 negativity (predictive of platinum response); 56% of TMB‐H also have MGMT negativity (predictive of dacarbazine and temozolomide response); 76% of TMB‐H also have RRM1 negative (predictive of gemcitabine response); 89% of TMB‐H patients have TOP2A positivity (predictive of with doxorubicin, epirubicin and etoposide response), 61% of TMB‐H patients have TOPO1 positivity (predictive of or irinotecan or topotecan response), 61% of TMB‐H patients have TS negativity (predictive of fluorouracil/pemetrexed/capecitabine response) and 29% of TMB‐H patients have TUBB3 negativity (predictive of better response to taxanes). (*c*) PD‐L1 positive (predictive of checkpoint inhibitor response) versus PD‐L1 negative. Seventy‐five percentage of PD‐L1 positive also have ERCC1 negativity (predictive of platinum response); 52% of PD‐L1 positive also have MGMT negativity (predictive of dacarbazine and temozolomide response); 74% of PD‐L1 positive also have RRM1 negative (predictive of gemcitabine response); 86% of PD‐L1 positive patients have TOP2A positivity (predictive of with doxorubicin, epirubicin, etoposide response), 58% of PD‐L1 positive patients have TOPO1 positivity (predictive of irinotecan or topotecan response), 49% of PD‐L1 positive patients have TS negativity (predictive of fluorouracil/pemetrexed/capecitabine response) and 38% of PD‐L1 positive patients have TUBB3 negativity (predictive of better response to taxanes). See Table 2 for additional data.

1 Bolded numbers indicated percentage of patients that may be responsive. No patients had all markers. See also Figure 1 for graphical presentation. See Materials and Methods section and Supporting Information Table S2 for the methods used in each case to determine positive or negative/low and Supporting Information Table S1 for the implication of positivity and negativity.

Abbreviations: ERCC1, excision repair complementation group 1; MGMT, O‐6‐methyl guanine DNA methyltransferase; MSI, microsatellite instability; RRM1, ribonucleotide reductase regulatory subunit M1; TMB, tumor mutational burden; TOP2A, topoisomerase 2; TOPO1, topoisomerase 1; TS, thymidylate synthase; TUBB3, tubulin beta 3.

Positivity of protein marker expression for all cancers combined was: ERCC1 20.9%, MGMT 55.4%, RRM1 19.9%, TOPO1 58.7%, TOP2A 75.8%, TS 34.0% and TUBB3 56.8% (Table 1 ). The percentage of protein expression positivity varied between cancer types (Supporting Information Table S4). For some of these proteins, for example, ERCC1, RRM1, MGMT, TS and TUBB3, it is loss of expression that correlates with either sensitivity or less resistance to chemotherapy.^23^, ^24^, ^25^, ^26^, ^27^, ^28^, ^29^, ^30^, ^31^, ^32^, ^33^, ^34^ Decreased expression for these proteins was found in the following patients by percent: ERCC1 79.1%, RRM1 80.1%, MGMT 44.6%, TS 66% and TUBB3 43.2%.

### MSI‐H and chemotherapy protein marker relationships

The relationship between the percentage of patients with protein expression indicating sensitivity to specific drugs was compared between MSI‐H and MSI‐Stable patients (Fig. 1 *a* and Table 2). The M‐H test was used to compare the likelihood of MSI‐H status with ERCC1, MGMT, RRM1, TOP2A, TOPO1, TS and TUBB3 expression indicating drug sensitivity (Table 2). Decreased ERCC1 expression, a marker of potential benefit from platinum chemotherapy,^29^, ^30^ was associated with MSI‐H status (M‐H OR [95% confidence interval {CI}]: 0.68 (0.55–0.85); *p* < 0.001). Similarly, low TUBB3 expression (high TUBB3 is a marker of taxane resistance^26^, ^27^, ^28^) was found more commonly in MSI‐H patients (M‐H OR 0.71 [0.60–0.83]; *p* < 0.001). Conversely, decreased TOPO1 expression (positivity is a marker for likely irinotecan or topotecan response^20^) was associated with MSI‐H; similarly, RRM1 overexpression (underexpression is a marker of gemcitabine response^34^) was more commonly found in with MSI‐H patients. No significant relationship was found between MSI status and MGMT expression (*p* = 0.59).

**Table 2:** Relationship between protein biomarkers and MSI‐H, TMB‐H or PD‐L1 expression status^1^

Biomarker	Mantel‐Haenszel odds ratio (95% CI)^2^	*p* Value	Number of patients	Potential benefit in combination therapy (yes/no)
*Protein markers and MSI‐H status*				
ERCC1	0.68 (0.55–0.85)	0.001	21,772	Yes for benefit of combination of immunotherapy and platinum (ERCC1 negativity, which is associated with platinum response,^29^, ^30^ was correlated with MSI‐H).
MGMT	0.91 (0.62–1.30)	0.59	5,175	No significant correlation between MGMT status and MSI‐H.
RRM1	3.49 (2.91–4.17)	<0.001	17,190	No for benefit of combination of immunotherapy and gemcitabine (RRM1 negativity is associated with gemcitabine response.^34^ However, data show that it is RRM1 positivity that was correlated with MSI‐H).
TOPO1	0.77 (0.67–0.89)	0.001	22,186	No for benefit of combination of immunotherapy and irinotecan or topotecan. (TOPO1 positivity is associated with irinotecan response.^20^ However, data show that TOPO1 negativity was correlated with MSI‐H).
TUBB3	0.71 (0.60–0.83)	<0.001	19,839	Yes for benefit of combination of immunotherapy and taxanes (TUBB3 positivity is associated with taxane resistance.^26^, ^27^, ^28^ Data shows that TUBB3 negativity was correlated with MSI‐H).
*Protein markers and TMB‐H status*				
ERCC1	0.83 (0.72–0.96)	0.013	21,665	Yes for benefit of combination of immunotherapy and platinum (ERCC1 negativity, which is associated with platinum response,^29^, ^30^ was correlated with TMB‐H).
MGMT	0.98 (0.80–1.20)	0.86	5,160	No significant correlation between MGMT status and TMB‐H.
TOP2A	2.80 (2.15–3.66)	<0.001	12,828	Yes for benefit of combination of immunotherapy and doxorubicin (TOPO2A positivity, which is associated with doxorubicin, etoposide, epirubicin response,^19^, ^21^ was correlated with TMB‐H).
TOPO1	0.83 (0.75–0.93)	0.001	22,090	No for benefit of combination of immunotherapy and irinotecan or topotecan (TOPO1 positivity is associated with irinotecan or topotecan response.^20^ However, data shows that it TOPO1 negativity was correlated with TMB‐H).
*Protein markers and PD‐L1 positivity*				
MGMT	0.78 (0.65–0.95)	0.011	4,919	Yes for benefit of combination of immunotherapy and temozolomide, dacarbazine. (MGMT negativity, which is associated with response to dacarbazine^31^ and temozolomide,^32^, ^33^ was correlated with PD‐L1 positivity).
TOPO1	1.01 (0.91–1.12)	0.87	18,931	No significant correlation between TOPO1 status and PD‐L1 positivity.

1 If odds ratio of biomarker is less than 1 and *p* value is significant, then biomarker negativity is associated with MSI‐H or TMB‐H. See summary of these results in Table 3.

2 Tumor types were pooled and described in this table by the Mantel‐Haenszel odds ratio if the Breslow‐Day test was not significant; if the Breslow‐Day test is significant, you cannot pool the tumor types because there are significant differences between histologies. Protein markers with significant Breslow‐Day results were not included in the table, and relationships are summarized in Supporting Information Tables S5–S7.

Abbreviations: CI, confidence interval; ERCC1, excision repair complementation group 1; H, high; MGMT, O‐6‐methyl guanine DNA methyltransferase; MSI, microsatellite instability; TMB, tumor mutational burden; TS, thymidylate synthase; TOPO1, topoisomerase 1; TUBB3, tubulin beta 3.

TS and TOP2A could not be evaluated by the M‐H test, which looks at pooled data for all histologies providing that the individual histologies do not differ significantly from each other. If the individual histologies differed significantly, we examined them with the Fisher's exact test. For TS, the Fisher's exact test was significant in 9 of 40 tumor types in the direction of drug resistance (Supporting Information Table S5). Specifically TS positivity (reflects 5‐fluoruracil resistance^22^) was associated with MSI‐H status in all nine tumor types (colorectal [*p* < 0.001], cholangiocarcinoma [*p* = 0.022], epithelial ovarian cancer [*p* = 0.031], female genital tract malignancy [*p* < 0.0001], gastric cancer [*p* < 0.001], neuroendocrine tumor [*p* = 0.025], cancer with unknown primary [*p* < 0.001], pancreatic [*p* < 0.001] and small intestinal cancers [*p* = 0.003]). For TOP2A, Fisher's exact tests were significant in 4 of 40 tumor types for drug sensitivity (Supporting Information Table S5). TOP2A positivity, a marker of potential doxorubicin, epirubicin and etoposide benefit,^19^, ^21^ was significantly associated with MSI‐H status in all four cancers (epithelial ovarian [*p* = 0.0018], female genital tract malignancy [*p* = 0.0011], gastric cancer [*p* = 0.043] and neuroendocrine tumors [*p* = 0.0038]).

### TMB‐H and chemotherapy protein marker relationships

The relationship between the percentage of patients with positive protein expression indicating sensitivity to specific drugs was compared between TMB‐H and TMB‐intermediate/‐low (Fig. 1 *b*). The M‐H test was used to compare the likelihood of TMB‐H status with ERCC1, MGMT, RRM1, TOP2A, TOPO1, TS and TUBB3 expression indicating drug sensitivity (Table 2). Decreased ERCC1 expression, a marker of potential benefit from platinum chemotherapy,^29^, ^30^ was associated with TMB‐H status (M‐H OR [95% CI]: 0.83 [0.72–0.96]; *p* = 0.013). TOP2A overexpression, a marker of doxorubicin, etoposide and epirubicin response,^19^, ^21^ was found more commonly in TMB‐H (M‐H OR 2.80 [2.15–3.66]; *p* < 0.001). No significant relationship was found between TMB status and MGMT expression (*p* = 0.86).

RRM1, TS and TUBB3 could not be evaluated by the M‐H test. For RRM1, the Fisher's exact test was significant in 9 of 40 tumor types (Supporting Information Table S6). RRM1 negativity, a marker of gemcitabine response,^34^ was associated with TMB‐H status in nonmelanoma skin cancer (*p* = 0.031). RRM1 positivity (negativity has been related to gemcitabine response^34^) was associated with TMB‐H status in eight tumor types: small intestinal cancer (*p* = 0.005), pancreatic cancer (*p* = 0.004), cancer with unknown primary (*p* = 0.010), nonsmall cell lung cancer (*p* = 0.012), female genital tract malignancy (*p* < 0.001), epithelial ovarian cancer (*p* < 0.001), breast cancer (*p* = 0.040) and bladder cancer (*p* = 0.019).

For TS, the Fisher's exact test was significant in 11 of 40 tumor types (Supporting Information Table S6). TS negativity, a marker for fluorouracil, pemetrexed and capecitabine response^23^, ^24^, ^25^ was associated with TMB‐H status in two tumor types: breast cancer (*p* = 0.029) and Merkel cell cancer (*p* = 0.048). TS positivity (negativity is associate with improved responses to 5‐flurouracil, pemetrexed and capecitabine^23^, ^24^, ^25^) was associated with TMB‐H status in nine tumor types: small intestinal cancer (*p* = 0.001), pancreatic cancer (*p* = 0.002), cancer with unknown primary tumors (*p* = 0.001), nonsmall cell lung cancer (*p* < 0.001), gastric cancer (*p* < 0.001), female genital tract malignancy (*p* < 0.001), epithelial ovarian cancer (*p* = 0.031), cholangiocarcinoma (*p* = 0.029) and colorectal cancer (*p* < 0.001).

For TUBB3, the Fisher's exact test was significant in 4 of 40 tumor types (Supporting Information Table S6). TUBB3 positivity, a marker for taxane resistance^26^, ^27^, ^28^ was associated with TMB‐H status in two tumor types: nonsmall cell lung cancer (*p* = 0.035) and melanoma (*p* < 0.001). TUBB3 negativity (positivity is associated with taxane resistance^26^, ^27^, ^28^) was associated with TMB‐H status in two tumor types: female genital tract malignancy (*p* = 0.039) and colorectal cancer (*p* = 0.046).

### PD‐L1 expression and chemotherapy protein marker relationships

The relationship between the percentage of patients with positive protein expression indicating drug sensitivity was compared between PD‐L1 expressing tumors and PD‐L1 nonexpressing tumors (Fig. 1 *c*). The M‐H test was used to compare the likelihood of PD‐L1 expression with ERCC1, MGMT, RRM1, TOP2A, TOPO1, TS and TUBB3 positivity (Table 2). Decreased MGMT expression, a marker for temozolomide and dacarbazine response,^31^, ^32^, ^33^ was found more commonly with PD‐L1 expression (M‐H OR [95% CI]: 0.78 [0.65–0.95]; *p* = 0.011). No relationship was found between TOPO1 and PD‐L1 expression (*p* = 0.87).

ERCC1, RRM1, TOP2A, TS and TUBB3 could not be evaluated by the M‐H test. For ERCC1, the Fisher's exact test was significant in 4 of 40 tumor types (Supporting Information Table S7). ERCC1 negativity, a marker of platinum response,^29^, ^30^ was associated with PD‐L1 expression in GIST tumors (*p* = 0.032), whereas ERCC1 positivity was associated with PD‐L1 expression in glioblastoma (*p* = 0.030), female genital tract malignancies (*p* < 0.001) and esophageal tumors (*p* = 0.010).

For RRM1, the Fisher's exact test was significant in 5 of 40 tumor types (Supporting Information Table S7). RRM1 positivity (RRM1 negativity has been associated with gemcitabine response^34^) was associated with PD‐L1 expression in nonepithelial ovarian cancer (*p* = 0.025), soft tissue sarcoma (*p* = 0.010), pancreatic cancer (*p* = 0.032), female genital tract malignancy (*p* = 0.016) and cholangiocarcinoma (*p* = 0.002).

For TOP2A, the Fisher's exact test was significant in 10 of 40 tumor types (Supporting Information Table S7). TOP2A positivity, a marker of doxorubicin, etoposide and epirubicin response,^19^, ^21^ was associated with PD‐L1 expression in nonepithelial ovarian cancer (*p* = 0.029), soft tissue sarcoma (*p* < 0.001), cancer with unknown primary (*p* < 0.001), nonmelanoma skin cancer (*p* = 0.045), nonsmall cell lung cancer (*p* < 0.001), neuroendocrine tumors (*p* = 0.003), mesothelioma (*p* = 0.013), kidney cancer (*p* < 0.001), head and neck cancer (*p* = 0.001) and female genital tract malignancy (*p* < 0.001).

For TS, the Fisher's exact test was significant in 15 of 40 tumor types (Supporting Information Table S7). TS positivity (TS negativity has been associated with fluorouracil, pemetrexed and capecitabine response^23^, ^24^, ^25^) was associated with PD‐L1 expression in small intestinal cancer (*p* = 0.007), pancreatic cancer (*p* < 0.001), cancer with unknown primary (*p* < 0.001), nonsmall cell lung cancer (*p* = 0.036), neuroendocrine tumors (*p* = 0.035), melanoma (*p* = 0.006), kidney cancer (*p* = 0.015), head and neck cancers (*p* < 0.001), gastric cancer (*p* < 0.001), female genital tract malignancy (*p* = 0.031), epithelial ovarian cancer (*p* = 0.008), cholangiocarcinoma (*p* = 0.006), colorectal cancer (*p* < 0.001), breast cancer (*p* < 0.001) and bladder cancer (*p* = 0.001).

For TUBB3, the Fisher's exact test was significant in 8 of 40 tumor types (Supporting Information Table S7). TUBB3 positivity, a marker for taxane resistance,^26^, ^27^, ^28^ was associated with PD‐L1 expression in soft tissue sarcoma (*p* = 0.027), cancer with unknown primary tumors (*p* = 0.019), nonsmall cell lung cancer (*p* < 0.001), kidney cancer (*p* = 0.006), head and neck cancer (*p* = 0.035), gastric cancer (*p* = 0.001), esophageal cancer (*p* = 0.004) and bladder cancer (*p* = 0.001).

## Discussion

Activating the immune system to fight metastatic malignancies has been a major breakthrough in cancer therapy particularly for melanoma and lung cancer. Given the heterogeneity and complexity of metastatic solid tumors,^38^, ^39^, ^40^, ^41^ it is important to give cancer therapy in combinations. Cytotoxic chemotherapy has the potential to augment the immune response and improve response rates and outcomes. However, cytotoxic chemotherapy can also have negative effects, including toxicities and immune cell depletion. It is unclear which chemotherapeutic agents would be most frequently effective when combined with checkpoint inhibitor immunotherapy.

The current study explored relationships between markers of chemotherapy response and of response to anti‐PD1/PD‐L1 immunotherapy, such as MSI‐H, TMB‐H and PD‐L1 expression.^11^, ^12^, ^14^ The overall findings are summarized in Table 3. ERCC1 negativity, a marker of platinum response,^29^, ^30^ was frequently correlated with both MSI‐H and TMB‐H status in the pooled analysis of tumors but was not correlated with PD‐L1 IHC‐positive status across tumor types. Overall, this would predict a potential benefit for immunotherapy and platinum agent combinations in patients with MSI‐H or TMB‐H. MGMT negativity correlated with PD‐L1 expression but was not significantly correlated with MSI‐H and TMB‐H evaluations. This would predict a potential benefit for dacarbazine or temozolomide^31^, ^32^, ^33^ combined with checkpoint inhibitors in patients whose tumors expressed PD‐L1 by IHC but not necessarily in those with MSI‐H or TMB‐H. RRM1 positivity (negativity is a biomarker for gemcitabine response^34^) was associated with MSI‐H. This relationship was also found for many tumor types with TMB‐H and PD‐L1, suggesting that gemcitabine would not benefit most of these patients in combination with checkpoint blockade immunotherapy. TOP2A positivity, which predicts response to doxorubicin, epirubicin and etoposide,^19^, ^21^ was associated with TMB‐H. This relationship was also found for many tumor types with MSI‐H and PD‐L1. TOPO1 negativity correlated with MSI‐H and TMB‐H, but TOPO1 levels were not significantly associated with PD‐L1 expression. As TOPO1 negativity suggests lack of response to irinotecan and topotecan (positivity is predictive of response^20^), these data indicate infrequent benefit from combining anti‐PD‐1/PD‐L1 agents with irinotecan or topotecan. TS positivity, a marker of attenuated response to fluorouracil, pemetrexed or capecitabine,^22^, ^23^, ^24^, ^25^ correlated with MSI‐H, TMB‐H and PD‐L1 in many tumor types; thus, combinations involving fluorouracil, pemetrexed or capecitabine and immunotherapy are less likely to be of benefit. TUBB3 negativity, a marker of taxane response,^26^, ^27^, ^28^ was associated with MSI‐H, whereas negativity (taxane resistance) was related to PD‐L1 in many tumor types; thus, the benefit for combining taxanes with immunotherapy is likely to be more frequent in patients with MSI‐H and less frequent in those with PD‐L1 expression (Table 3).

**Table 3:** Summary of benefit for immunotherapy and chemotherapy combinations^1^

Marker^2^	MSI‐H	TMB‐H	PD‐L1 expression
**ERCC1** (platinum)	**MSI‐H and ERCC1 negative are associated; therefore**, **immunotherapy and platinum combinations are likely beneficial**.	**TMB‐H and ERCC1 negative are associated; therefore**, **immunotherapy and platinum combination are likely beneficial**.	PD‐L1 expression and ERCC1 positive were associated in 3 of 39 tumor types; hence, combinations of platinum and immunotherapy are not beneficial. In 1 of 39 tumor types, PD‐L1 expression and ERCC1 negative were associated; hence, combinations of platinum and immunotherapy are likely beneficial in this tumor type. In the other 35 tumor types, there was no relationship between PDL1 expression and ERCC1.
**MGMT** (dacarbazine/temozolomide)	No significant associations between MGMT negative and MSI‐H.	No significant associations between MGMT negative and TMB‐H.	**MGMT decreased was associated with PD‐L1 expression**. **Hence**, **the combination of dacarbazine or temozolomide with immunotherapy is likely of benefit**.
**RRM1** (gemcitabine)	MSI‐H was associated with RRM1 positivity. Hence, gemcitabine and immunotherapy is unlikely to be of benefit.	TMB‐H was associated with RRM1 positivity in 8 of 40 tumor types and hence the combination of immunotherapy and gemcitabine is unlikely to be of benefit in these tumor types. TMB‐H was associated with RRM1 negativity in 1 of 40 tumor types and hence the combination of immunotherapy and gemcitabine is likely to be of benefit in this tumor types. In the other 31 tumor types, there was no relationship between TMB‐H and RRM1.	PD‐L1 expression was associated with RRM1 positivity in 5 out of 40 tumor types; hence, gemcitabine and immunotherapy is unlikely to be of benefit. In the other 35 tumor types, there was no relationship between PD‐L1 expression and RRM1.
**TOP2A (**doxorubicin/etoposide/epirubicin)	MSI‐H was associated with TOP2A positivity in 4 of 40 tumor types; hence, doxorubicin/etoposide/epirubicin and immunotherapy is likely to be of benefit. In the other 36 tumor types, there was no relationship between MSI‐H and TOP2A.	**TMB‐H and TOP2A positivity are associated; therefore**, **immunotherapy and doxorubicin/etoposide/epirubicin combination are likely beneficial.**	**PD‐L1 expression was associated with TOP2A positivity in 10 of 40 tumor types; hence**, **doxorubicin/etoposide/epirubicin and immunotherapy is likely to be of benefit**. **In the other 30 tumor types**, **there was no relationship between PD‐L1 expression and TOP2A**.
**TOPO1** (irinotecan/topotecan)	MSI‐H was associated with TOPO1 negativity. Hence, irinotecan/topotecan and immunotherapy is unlikely to be of benefit.	TMB‐H was associated with TOPO1 negativity. Hence, irinotecan and immunotherapy is unlikely to be of benefit.	No significant associations between TOPO1 positive and PD‐L1 expression.
**TS** (fluorouracil/pemetrexed/capecitabine)	MSI‐H was associated with TS positivity in 9 of 40 tumor types; hence, fluorouracil/pemextrexed/capecitabine and immunotherapy is unlikely to be of benefit. In the other 31 tumor types, there was no relationship between PDL1 expression and TS.	TMB‐H expression and TS positive were associated in 9 of 40 tumor types; hence, combinations of fluoruracil/pemetrexed/capecitabine and immunotherapy are not beneficial In 2 of 40 tumor types, TMB‐H and TS positive were associated; hence, combinations of fluorouacil/pemetrexed/capecitabine and immunotherapy are likely beneficial. In the other 29 tumor types, there was no relationship between TMB‐H expression and TS.	PD‐L1 expression was associated with TS positivity in 15 of 40 tumor types; hence, fluorouracil/pemextrexed/capecitabine and immunotherapy is unlikely to be of benefit. In the other 25 tumor types, there was no relationship between PDL1 expression and TS.
**TUBB3** (taxane)	**MSI‐H and TUBB3 negative are associated; therefore**, **immunotherapy and doxorubicin/etoposide/epirubicin combination are likely beneficial**.	TMB‐H and TUBB3 positive were associated in 2 of 40 tumor types; hence, combinations of taxanes and immunotherapy are not beneficial. In 2 of 40 tumor types, TMB‐H and TUBB3 negative were associated; hence, combinations of taxanes and immunotherapy are likely beneficial in this tumor type. In the other 36 tumor types, there was no relationship between TMB‐H and TUBB3.	PD‐L1 expression was associated with TUBB3 positivity in 8 of 40 tumor types; hence, taxanes and immunotherapy is unlikely to be of benefit. In the other 32 tumor types, there was no relationship between PDL1 expression and TUBB3.

See also Supporting Information Table S1 for biomarker‐chemotherapy relationships. Bold “benefit” signifies the strongest evidence to for benefit of immunotherapy and chemotherapy combination.

1 Summary from Mantel‐Haenszel tests that pools data from the 40 histologies tested (Table 2). For patients where Mantel‐Haenszel could not be used (i.e., different histologies showed different results), number of tumor types with significant results are listed—in that case, the Fisher exact test was used to calculate significant associations; see Supporting Information Tables S5–S7 for the exact tumor types in each case.

2 For ERCC1, MGMT, RRM1, TS and TUBB3, it is low or negative expression of the marker that is associated with chemotherapy benefit; for TOP2A and TOPO1, it is positive expression that is associated with chemotherapy benefit. Therefore, as examples, in this table, ERCC1 negativity (potential benefit from platinums) is associated with MSI‐H (potential immunotherapy benefit) and TOP2A positivity (potential benefit from doxorubicin, etoposide and epirubicin) is associated with MSI‐H (potential benefit from immunotherapy) in 4 of 40 tumor types.

Abbreviations: ERCC1, excision repair complementation group 1; MGMT, O‐6‐methyl guanine DNA methyltransferase; MSI, microsatellite instability; PD‐L1, programmed death‐ligand 1; RRM1, ribonucleotide reductase regulatory subunit M1; TMB, tumor mutational burden; TOP2A, topoisomerase 2; TOPO1, topoisomerase 1; TS, thymidylate synthase; TUBB3, tubulin beta 3.

Prior studies of the relationships between protein markers and response to cytotoxic chemotherapy were performed in a disease‐specific manner. These studies evaluated commonly used chemotherapeutic agents for each cancer type. However, these relationships may hold for other cancer types.^21^, ^42^ In our study, although many of the statistical relationships determined were valid across tumor types, most of the PD‐L1 assessments and all TS relationships were evaluated by individual tumor type as the significant differences between tumor types did not allow for pooling. Thus, any relationships found only held for a subset of the tumor types evaluated. In some cases, the lack of significance for other tumor types may be due to lack of power for the individual tumor types.

Prior oncology therapeutics and regimens were approved by the Food and Drug Administration (FDA) and administered based on tissue of origin of the tumor. However, recent advances in NGS and molecular profiling have demonstrated that each tumor has a unique molecular profile, which mandates a more personalized approach.^39^, ^40^, ^43^ Recent studies have explored dosing of novel combinations of targeted agents, cytotoxics and immunotherapies.^3^, ^44^, ^45^, ^46^ The FDA recently approved pembrolizumab in a tissue‐agnostic manner for use in all patients with MSI‐H status or mismatch gene alterations,^47^ which signifies a major shift in drug approval practice. Additional studies have suggested that patients with high TMB^12^ or PD‐L1 expression^14^ will have superior responses to checkpoint blockade immunotherapy. Although cytotoxic chemotherapy has also traditionally been administered based on tumor of origin, more recent data^19^, ^20^, ^21^, ^22^, ^23^, ^24^, ^25^, ^26^, ^27^, ^28^, ^29^, ^30^, ^31^, ^32^, ^33^, ^34^ support the use of protein markers to provide insight into how best to match these agents to an individual patient.

All patients would ideally have molecular profiling including MSI, TMB, PD‐L1 and protein marker information prior to the start of therapy, but due to delays in acquiring tissue from pathology and conducting NGS, patients may not have a full genomic and protein marker profile at the start of treatment. This is especially true for a patient who needs urgent initiation of therapy due to organ failure from malignancy. Immunotherapy and chemotherapy combinations are increasingly being studied in clinical trials; thus, a better understanding of the relationships between markers of response to chemotherapy and immunotherapy—in particular, which combinations will give a higher probability of response—as evaluated in the current study, is essential.

Other findings of interest also emerged from out interrogation of tissue markers. MSI‐H status was only observed in 3%, whereas TMB‐H was seen in 8.4% of the patients reviewed and PD‐L1 expression was present in 11.0% patients in the current study. Other studies have shown corresponding percentages of 7.1% (TMB‐H)^48^ and 3.5% (MSI‐H).^49^ In the current study, only 28% of patients with TMB‐H status had concurrent MSI‐H; however, the majority of patients with MSI‐H status were TMB‐H (73%). A prior study of TMB across tumor types showed that 83% of patients with MSI‐H status had TMB‐H; however, only 16% of TMB‐H patients had MSI‐H status.^48^ Given that MSI‐H, TMB‐H and PD‐L1 expression often do not cooccur, it is not surprising that the current study often found distinct relationships between each of these three immunotherapy markers and the protein markers.

This study had several important limitations. First, the database was deidentified; hence, future studies will need to determine if these relationships correlate with better outcomes for the cognate combinations. Some markers can be evaluated by more than one methodology, and precise‐points for important markers such as TMB‐H are still a matter of debate. Third, the markers of response to chemotherapy were assumed to hold for all tumor types; however, they may not have been validated in all tumor types. Of note, ERCC1 has not been found to be predictive of nonsmall cell lung cancer responses to platinum agents^50^; thus, we did not evaluate ERCC1 relationships with MSI, TMB and PD‐L1 in nonsmall cell lung cancer. Furthermore, a significant M‐H test indicates that a relationship exists between immunotherapy and chemotherapy response markers when taking into account possible confounding from the different tumor types, but does not mean there is a relationship present for each individual tumor type. The study aimed to make overall conclusions regarding immunotherapy and chemotherapy response marker associations to provide clinically useful information to help guide precision medicine treatments and clinical trials. Fourth, the mechanisms underlying the associations described in this report are not clear. Fifth, specific treatments might result in a change in expression of PD‐L1 or other markers as the tumor evolves. Finally, the ability of protein markers to predict chemotherapy response is still not considered as robust as the predictive power of NGS for immunotherapy or gene‐targeted agents.

In conclusion, MSI‐H, TMB‐H and PD‐L1 expressions were found to correlate with specific protein markers of response to chemotherapy. Based on the cooccurrence of these biomarkers, combinations of PD1/PD‐L1 checkpoint inhibitor immunotherapy with temozolomide, dacarbazine, doxorubicin, epirubicin, etoposide and platinum will have a higher probability of a response, whereas combinations of immunotherapy with irinotecan, topotecan, gemcitabine, fluorouracil, pemetrexed and capecitabine may less frequently have salutary effects. Taxanes may be of more frequent benefit to patients with MSI‐H but not those with TMB‐H or PD‐L1 expression. Protein markers of chemotherapy response along with NGS for immunotherapy response markers should be evaluated in prospective trials to determine if these markers can help support the rational use of chemotherapy as part of an individualized, precision medicine approach to oncology therapy.

## Supporting information


**Appendix S1:** Supporting information
