# Supplementary material for: Relationship between protein biomarkers of chemotherapy response and microsatellite status, tumor mutational burden and PD‐L1 expression in cancer patients
Source: Int J Cancer. 2019 Oct 1;146(11):3087–97. doi: 10.1002/ijc.32661 (PMC7051881; doi:10.1002/ijc.32661)
Supplement: Supplementary file 1 — Appendix S1: Supporting information [file IJC-146-3087-s001.doc]

**Supplemental Table 1:** Relationship between protein expression and potential chemotherapy benefit

|  | **Protein Expression** | **Chemotherapy of Potential Benefit** |
| --- | --- | --- |
| **ERCC1** | Negative | Platinum |
| **MGMT** | Negative | Dacarbazine, Temozolomide |
| **RRM1** | Negative | Gemcitabine |
| **TOP2A** | Positive | Doxorubicin, Epirubicin, Etoposide |
| **TOPO1** | Positive | Irinotecan, Topotecan |
| **TS** | Negative | Fluorouracil, Pemetrexed |
| **TUBB3** | Negative | Taxanes* |

*TUBB3 positivity predicts taxane resistance

Abbreviations: ERCC1=excision repair complementation group 1, MGMT= O-6-methyl guanine DNA methyltransferase, RRM1=ribonucleotide reductase regulatory subunit M1, TOP2A=topoisomerase 2, TOPO1=topoisomerase 1, TS=thymidylate synthetase, TUBB3=tubulin beta 3

**Supplemental Table 2: Methodology for determining various markers shown in Table 1***

|  | **Method** | **Comment** |
| --- | --- | --- |
| **MSI-H** | NGS | Direct analysis of short tandem repeat tracts in the target regions of sequenced genes; High: ≥46 altered microsatellite loci** |
| **TMB-H** | NGS | Calculated using nonsynonymous missense mutations; common germline variants excluded; High TMB:  ≥17 mutations/Mb** |
| **PD-L1** | IHC | Antibody: SP142 (Ventana); Tumor tissue staining with ≥5% considered positive** |
| **ERCC1** | IHC | Antibody: 8F1 (Abcam); Positive: staining intensity of 2+ in at least 50% or 3+ in at least 10% or more tumor cells on a given slide |
| **MGMT** | IHC | Antibody: MT21.2 (Invitrogen); Positive: staining intensity of ≥ 2+ in at least 50% or more tumor cells on a given slide |
| **RRM1** | IHC | Antibody: polyclonal (Proteintech Group); Positive: staining intensity of 2+ or 3+ in at least 35% or more tumor cells on a given slide |
| **TOP2A** | IHC | Antibody: 3F6 (Leica Microsystems); Positive: staining intensity of ≥1+ in at least 10% or more tumor cells on a given slide |
| **TOPO1** | IHC | Antibody: 1D6 (Leica Microsystems); Positive: staining intensity of 2+ or 3+ in at least 30% or more tumor cells on a given tumor section |
| **TS** | IHC | Antibody: TS106 (Dako); Positive: staining intensity of ≥1+ in at least 10% or more tumor cells on a given slide |
| **TUBB3** | IHC | Antibody: PRB-435P (BioLegend); Positive: staining intensity of 2+ or 3+ in at least 30% or more tumor cells on a given tumor section |

*See also **Methods**

**Gatalica Z, Xiu J, Swensen J, et al: Comprehensive analysis of cancers of unknown primary for the biomarkers of response to immune checkpoint blockade therapy. Eur J Cancer 94:179-186, 2018

**Abbreviations:** ERCC1=excision repair complementation group 1, MGMT= O-6-methyl guanine DNA methyltransferase, MSI =microsatellite instability, NGS = next generation sequencing; RRM1=ribonucleotide reductase regulatory subunit M1, TMB = tumor mutational burden; TOP2A=topoisomerase 2, TOPO1=topoisomerase 1, TS=thymidylate synthetase, TUBB3=tubulin beta 3

**Supplemental Table 3: MSI, TMB, PD-L1 by Cancer Type (bolded percentages are indicative of response to cognate therapy)***

|  | **MSI-H**** | | | **TMB-H**** | | | **PD-L1**** | | |
| --- | --- | --- | --- | --- | --- | --- | --- | --- | --- |
| **Type of Cancer** | **N** | **Positive** | **Negative** | **N** | **Positive** | **Negative** | **N** | **Positive** | **Negative** |
| All | 28034 | **3.3%** | 96.7% | 27847 | **8.4%** | 91.6% | 22114 | **11.0%** | 89.0% |
| Adrenal | 40 | **0.0%** | 100.0% | 40 | **2.5%** | 97.5% | 39 | **12.8%** | 87.2% |
| Anal | 101 | **0.0%** | 100.0% | 100 | **6.0%** | 94.0% | 99 | **40.4%** | 59.6% |
| Bladder | 298 | **0.7%** | 99.3% | 297 | **17.8%** | 82.2% | 294 | **25.2%** | 74.8% |
| Bone | 77 | **1.3%** | 98.7% | 77 | **1.3%** | 98.7% | 74 | **0.0%** | 100.0% |
| Breast | 2427 | **0.7%** | 99.3% | 2397 | **3.6%** | 96.4% | 2293 | **5.8%** | 94.2% |
| CNS tumors | 171 | **0.0%** | 100.0% | 171 | **1.2%** | 98.8% | 171 | **8.8%** | 91.2% |
| Colorectal cancer | 3848 | **6.3%** | 93.7% | 3831 | **7.3%** | 92.7% | 3615 | **3.5%** | 96.5% |
| Cholangiocarcinoma | 434 | **1.8%** | 98.2% | 426 | **3.5%** | 96.5% | 400 | **9.0%** | 91.0% |
| Epithelial Ovarian | 3855 | **1.3%** | 98.7% | 3818 | **1.7%** | 98.3% | 3674 | **6.7%** | 93.3% |
| Esophageal | 532 | **1.3%** | 98.7% | 527 | **2.5%** | 97.5% | 497 | **12.5%** | 87.5% |
| Extrahepatic cholangiocarcinoma | 68 | **1.5%** | 98.5% | 67 | **1.5%** | 98.5% | 65 | **3.1%** | 96.9% |
| Female genital tract malignancy | 2874 | **14.3%** | 85.7% | 2854 | **11.6%** | 88.4% | 2777 | **10.2%** | 89.8% |
| GBM | 948 | **0.6%** | 99.4% | 943 | **3.0%** | 97.0% | 910 | **15.2%** | 84.8% |
| GIST | 154 | **0.0%** | 100.0% | 154 | **0.0%** | 100.0% | 179 | **19.0%** | 81.0% |
| Gastric | 495 | **7.7%** | 92.3% | 492 | **7.5%** | 92.5% | 459 | **10.2%** | 89.8% |
| HCC | 179 | **1.7%** | 98.3% | 177 | **2.8%** | 97.2% | 174 | **7.5%** | 92.5% |
| Head and Neck | 526 | **0.6%** | 99.4% | 523 | **6.5%** | 93.5% | 494 | **36.6%** | 63.4% |
| Kidney | 353 | **0.6%** | 99.4% | 349 | **8.6%** | 91.4% | 343 | **19.8%** | 80.2% |
| Low Grade Glioma | 193 | **0.5%** | 99.5% | 193 | **1.0%** | 99.0% | 176 | **2.8%** | 97.2% |
| Merkel cell | 12 | **0.0%** | 100.0% | 12 | **41.7%** | 58.3% | 12 | **0.0%** | 100.0% |
| MFST | 9 | **0.0%** | 100.0% | 9 | **0.0%** | 100.0% | 8 | **12.5%** | 87.5% |
| Male Genital Tract | 56 | **0.0%** | 100.0% | 56 | **5.4%** | 94.6% | 55 | **12.7%** | 87.3% |
| Melanoma | 812 | **0.0%** | 100.0% | 811 | **37.5%** | 62.5% | 800 | **23.2%** | 76.8% |
| Mesothelioma | 99 | **0.0%** | 100.0% | 99 | **0.0%** | 100.0% | 96 | **24.0%** | 76.0% |
| NET | 463 | **2.2%** | 97.8% | 462 | **5.0%** | 95.0% | 437 | **4.8%** | 95.2% |
| NSCLC | 4754 | **0.7%** | 99.3% | 4752 | **15.9%** | 84.1% | 822 | **30.2%** | 69.8% |
| Non-melanoma skin | 143 | **2.1%** | 97.9% | 143 | **66.4%** | 33.6% | 141 | **34.8%** | 65.2% |
| Cancer with unknown primary | 788 | **3.0%** | 97.0% | 779 | **11.9%** | 88.1% | 761 | **22.6%** | 77.4% |
| Other | 11 | **0.0%** | 100.0% | 11 | **0.0%** | 100.0% | 11 | **9.1%** | 90.9% |
| Pancreatic | 1261 | **1.4%** | 98.6% | 1248 | **1.4%** | 98.6% | 1174 | **10.8%** | 89.2% |
| Peritoneal | 17 | **0.0%** | 100.0% | 17 | **11.8%** | 88.2% | 17 | **23.5%** | 76.5% |
| Prostate | 463 | **3.7%** | 96.3% | 456 | **3.7%** | 96.3% | 443 | **3.4%** | 96.6% |
| Retroperitoneal sarcoma | 115 | **0.0%** | 100.0% | 113 | **0.0%** | 100.0% | 107 | **11.2%** | 88.8% |
| SCLC | 220 | **0.5%** | 99.5% | 220 | **6.4%** | 93.6% | 191 | **4.7%** | 95.3% |
| Soft tissue sarcoma | 613 | **1.0%** | 99.0% | 607 | **4.1%** | 95.9% | 573 | **13.4%** | 86.6% |
| Small intestinal | 218 | **10.1%** | 89.9% | 214 | **7.9%** | 92.1% | 205 | **12.2%** | 87.8% |
| Thymic | 52 | **3.8%** | 96.2% | 52 | **1.9%** | 98.1% | 49 | **73.5%** | 26.5% |
| Thyroid | 108 | **1.9%** | 98.1% | 108 | **0.1%** | 99.9% | 99 | **33.3%** | 66.7% |
| Uveal melanoma | 112 | **0.9%** | 99.1% | 111 | **2.7%** | 97.3% | 105 | **4.8%** | 95.2% |
| Non-Epithelial Ovarian | 135 | **0.7%** | 99.3% | 132 | **0.8%** | 99.2% | 127 | **3.1%** | 96.9% |
|  |  |  |  |  |  |  |  |  |  |
|  |  |  |  |  |  |  |  |  |  |

*Percentage of patients positive for the marker

** PD-L1 positivity as well as MSI-H and TMB-H is associated with sensitivity to checkpoint inhibitors (therefore, for all cancers, 3.3%, 8.4 % and 11.0% of patients, respectively, would be expected to be responsive.

**Abbreviations:** CNS=central nervous system; GIST=gastrointestinal stromal tumor; GBM=glioblastoma multiforme; HCC=hepatocellular carcinoma; MFST=malignant solitary fibrous tumor of the pleura; MSI =microsatellite instability, NET=neuroendocrine tumor; NSCLC=non-small cell lung cancer; PD-L1=programmed death-ligand 1, SCLC=small cell lung cancer; TMB = tumor mutational burden

**Supplemental Table 4: Protein Expression by Cancer Type (bolded percentages are indicative of response to cognate therapy)***

|  | **ERCC1**** | | | **MGMT**** | | | **RRM1**** | | | **TOP2A**** | | | **TOPO1**** | | | **TS**** | | | **TUBB3**** | | |
| --- | --- | --- | --- | --- | --- | --- | --- | --- | --- | --- | --- | --- | --- | --- | --- | --- | --- | --- | --- | --- | --- |
| **Type of Cancer** | **N** | **+** | **-** | **N** | **+** | **-** | **N** | **+** | **-** | **N** | **+** | **-** | **N** | **+** | **-** | **N** | **+** | **-** | **N** | **+** | **-** |
| All | 21802 | 21% | **79%** | 5200 | 55% | **45%** | 17205 | 20% | **80%** | 12907 | **76%** | 24% | 22211 | **59%** | 41% | 20491 | 34% | **66%** | 19863 | 57% | **43%** |
| Adrenal | 39 | 41% | **59%** | 9 | 44% | **56%** | 33 | 30% | **70%** | 39 | **69%** | 31% | 14 | **43%** | 57% | 39 | 67% | **33%** | 39 | 39% | **62%** |
| Anal | 97 | 32% | **68%** | 12 | 58% | **42%** | 77 | 47% | **53%** | 99 | **97%** | 3% | 32 | **56%** | 44% | 99 | 64% | **36%** | 99 | 38% | **62%** |
| Bladder | 290 | 20% | **80%** | 43 | 54% | **47%** | 266 | 26% | **74%** | 293 | **93%** | 7% | 85 | **47%** | 53% | 292 | 46% | **54%** | 292 | 51% | **49%** |
| Bone | 76 | 40% | **61%** | 65 | 75% | **25%** | 69 | 20% | **80%** | 74 | **55%** | 45% | 71 | **58%** | 42% | 42 | 48% | **52%** | 73 | 47% | **53%** |
| Breast | 2300 | 28% | **72%** | 336 | 73% | **27%** | 1058 | 29% | **71%** | 0 | **.** | . | 1911 | **69%** | 31% | 1957 | 33% | **67%** | 0 | . | **.** |
| CNS tumors | 169 | 25% | **75%** | 27 | 70% | **30%** | 111 | 14% | **86%** | 167 | **37%** | 64% | 77 | **38%** | 62% | 167 | 34% | **66%** | 168 | 52% | **48%** |
| Colorectal cancer | 3506 | 18% | **82%** | 1133 | 60% | **41%** | 296 | 37% | **63%** | 532 | **92%** | 9% | 3502 | **59%** | 41% | 3664 | 24% | **76%** | 529 | 45% | **55%** |
| Cholangio-carcinoma | 392 | 27% | **73%** | 68 | 65% | **35%** | 379 | 8% | **92%** | 84 | **64%** | 36% | 390 | **67%** | 33% | 400 | 29% | **72%** | 397 | 58% | **42%** |
| Epithelial Ovarian | 3664 | 12% | **88%** | 200 | 79% | **22%** | 3683 | 16% | **84%** | 3751 | **77%** | 23% | 3629 | **52%** | 48% | 632 | 51% | **49%** | 3751 | 50% | **50%** |
| Esophageal | 506 | 36% | **64%** | 46 | 54% | **46%** | 30 | 33% | **67%** | 513 | **96%** | 4% | 500 | **79%** | 21% | 513 | 43% | **57%** | 513 | 48% | **52%** |
| Extrahepatic cholangiocarcinoma | 65 | 17% | **83%** | 8 | 100% | **0%** | 62 | 7% | **94%** | 10 | **70%** | 30% | 60 | **52%** | 48% | 65 | 25% | **75%** | 66 | 49% | **52%** |
| Female genital tract malignancy | 2693 | 13% | **87%** | 156 | 44% | **56%** | 2767 | 31% | **69%** | 2817 | **87%** | 13% | 2641 | **47%** | 53% | 2803 | 53% | **47%** | 2820 | 57% | **44%** |
| GBM | 915 | 22% | **79%** | 44 | 25% | **75%** | 90 | 23% | **77%** | 139 | **63%** | 37% | 899 | **58%** | 42% | 405 | 52% | **48%** | 400 | 91% | **10%** |
| GIST | 80 | 48% | **53%** | 41 | 73% | **27%** | 26 | 12% | **89%** | 41 | **34%** | 66% | 43 | **37%** | 63% | 44 | 43% | **57%** | 41 | 44% | **56%** |
| Gastric | 473 | 28% | **72%** | 74 | 66% | **34%** | 54 | 26% | **74%** | 480 | **81%** | 19% | 466 | **68%** | 32% | 482 | 36% | **64%** | 483 | 32% | **69%** |
| HCC | 169 | 24% | **76%** | 28 | 61% | **39%** | 126 | 6% | **94%** | 168 | **51%** | 49% | 66 | **49%** | 52% | 171 | 26% | **74%** | 170 | 13% | **87%** |
| Head and Neck | 494 | 36% | **64%** | 62 | 68% | **32%** | 468 | 33% | **67%** | 200 | **68%** | 32% | 88 | **56%** | 44% | 503 | 40% | **60%** | 504 | 44% | **56%** |
| Kidney | 335 | 24% | **76%** | 61 | 54% | **46%** | 317 | 4% | **96%** | 347 | **37%** | 63% | 127 | **43%** | 57% | 134 | 21% | **79%** | 345 | 50% | **50%** |
| Low Grade Glioma | 183 | 13% | **87%** | 6 | 17% | **83%** | 9 | 0% | **100%** | 18 | **0%** | 100% | 181 | **37%** | 64% | 76 | 5% | **95%** | 76 | 76% | **24%** |
| MC | 12 | 8% | **92%** | 2 | 50% | **50%** | 6 | 83% | **17%** | 12 | **100%** | 0% | 10 | **80%** | 20% | 7 | 71% | **29%** | 6 | 100% | **0%** |
| MFST | 8 | 13% | **88%** | 8 | 88% | **13%** | 7 | 14% | **86%** | 8 | **50%** | 50% | 8 | **38%** | 63% | 2 | 100% | **0%** | 8 | 25% | **75%** |
| Male Genital Tract | 53 | 19% | **81%** | 8 | 50% | **50%** | 40 | 35% | **65%** | 54 | **82%** | 19% | 20 | **55%** | 45% | 54 | 44% | **56%** | 54 | 52% | **48%** |
| Melanoma | 794 | 26% | **74%** | 798 | 38% | **62%** | 31 | 13% | **87%** | 84 | **71%** | 29% | 81 | **44%** | 56% | 233 | 68% | **32%** | 803 | 69% | **31%** |
| Mesothelioma | 94 | 38% | **62%** | 19 | 58% | **42%** | 79 | 15% | **85%** | 95 | **39%** | 61% | 34 | **65%** | 35% | 95 | 34% | **66%** | 96 | 67% | **33%** |
| NET | 435 | 12% | **88%** | 344 | 45% | **55%** | 79 | 25% | **75%** | 441 | **52%** | 48% | 158 | **53%** | 48% | 445 | 27% | **74%** | 182 | 76% | **24%** |
| NSCLC | 0 | . | **.** | 327 | 64% | **36%** | 4074 | 15% | **85%** | 368 | **79%** | 22% | 4102 | **66%** | 34% | 4233 | 25% | **75%** | 4236 | 71% | **30%** |
| Non-melanoma skin | 139 | 34% | **66%** | 14 | 36% | **64%** | 105 | 34% | **66%** | 141 | **85%** | 15% | 46 | **48%** | 52% | 141 | 42% | **58%** | 141 | 71% | **29%** |
| Cancer with unknown primary | 751 | 28% | **72%** | 85 | 60% | **40%** | 726 | 23% | **78%** | 256 | **78%** | 22% | 726 | **70%** | 30% | 764 | 33% | **67%** | 759 | 62% | **38%** |
| Other | 11 | 27% | **73%** | 4 | 50% | **50%** | 8 | 0% | **100%** | 11 | **36%** | 64% | 5 | **40%** | 60% | 10 | 40% | **60%** | 11 | 18% | **82%** |
| Pancreatic | 1161 | 26% | **75%** | 154 | 81% | **19%** | 1142 | 10% | **90%** | 184 | **65%** | 35% | 1141 | **58%** | 42% | 1189 | 18% | **82%** | 1180 | 60% | **41%** |
| Peritoneal | 17 | 0% | **100%** | 1 | 0% | **100%** | 16 | 31% | **69%** | 17 | **77%** | 24% | 14 | **36%** | 64% | 6 | 67% | **33%** | 17 | 77% | **24%** |
| Prostate | 442 | 18% | **82%** | 34 | 53% | **47%** | 25 | 28% | **72%** | 150 | **47%** | 53% | 36 | **69%** | 31% | 40 | 23% | **78%** | 446 | 26% | **74%** |
| Retroperitoneal sarcoma | 98 | 36% | **64%** | 97 | 46% | **54%** | 99 | 11% | **89%** | 99 | **67%** | 33% | 95 | **45%** | 55% | 29 | 41% | **59%** | 99 | 32% | **68%** |
| SCLC | 201 | 10% | **90%** | 171 | 25% | **75%** | 20 | 60% | **40%** | 206 | **97%** | 3% | 62 | **69%** | 31% | 210 | 50% | **51%** | 62 | 87% | **13%** |
| Soft tissue sarcoma | 574 | 30% | **70%** | 558 | 54% | **46%** | 553 | 13% | **87%** | 586 | **64%** | 36% | 570 | **51%** | 49% | 235 | 50% | **50%** | 584 | 53% | **47%** |
| Small intestinal | 200 | 18% | **83%** | 35 | 66% | **34%** | 88 | 16% | **84%** | 125 | **80%** | 20% | 141 | **52%** | 48% | 206 | 37% | **63%** | 128 | 45% | **55%** |
| Thymic | 49 | 16% | **84%** | 11 | 82% | **18%** | 42 | 21% | **79%** | 49 | **84%** | 16% | 18 | **56%** | 44% | 49 | 49% | **51%** | 49 | 20% | **80%** |
| Thyroid | 99 | 23% | **77%** | 13 | 62% | **39%** | 14 | 14% | **86%** | 99 | **42%** | 58% | 16 | **31%** | 69% | 18 | 17% | **83%** | 17 | 88% | **12%** |
| Uveal melanoma | 90 | 46% | **54%** | 91 | 52% | **48%** | 1 | 0% | **100%** | 21 | **33%** | 67% | 21 | **48%** | 52% | 16 | 38% | **63%** | 91 | 40% | **60%** |
| Non-Epithelial Ovarian | 128 | 14% | **86%** | 7 | 43% | **57%** | 129 | 21% | **79%** | 129 | **42%** | 58% | 125 | **54%** | 46% | 21 | 71% | **29%** | 128 | 32% | **68%** |

*Percentage of patients positive for the protein marker

** ERCC1 negativity is associated with platinum response (therefore, for all cancers, 79% of patients would be expected to be responsive) (1,2); MGMT negativity which is associated with response to dacarbazine(3) and temozolomide (4,5) (therefore, for all cancers, 45% of patients would be expected to be responsive); RRM1 negativity is associated with gemcitabine response (therefore, for all cancers, 80% of patients would be expected to be responsive) (6); TOPO2A positivity which is associated with doxorubicin response (therefore, for all cancers, 76% of patients would be expected to be responsive) (7); TOPO1 positivity is associated with irinotecan or topotecan response (therefore, for all cancers, 59% of patients would be expected to be responsive) (8) TS negativity is associated with response to fluorouracil/pemetrexed/capecitabine (therefore, for all cancers, 66% of patients would be expected to be responsive) (9-12); TUBB3 positivity is associated with taxane resistance (therefore, for all cancers, 43% of patients would be expected to be responsive) (13-15)

**Abbreviations:** CNS=central nervous system; ERCC1=excision repair complementation group 1, GIST=gastrointestinal stromal tumor; GBM=glioblastoma multiforme; HCC = hepatocellular carcinoma; MFST=malignant solitary fibrous tumor of the pleura; MGMT= O-6-methyl guanine DNA methyltransferase, NET=neuroendocrine tumor; NSCLC=non-small cell lung cancer; RRM1=ribonucleotide reductase regulatory subunit M1; SCLC = small cell lung cancer; TOP2A=topoisomerase 2; TOPO1=topoisomerase 1; TS=thymidylate synthetase; TUBB3=tubulin beta 3

**Supplemental Table 5: Relationship between MSI-H and protein markers***

| **Tumor type** | **Odds Ratio**  **(95% CI)** | **p-value** | **N** | **Comment** |
| --- | --- | --- | --- | --- |
| **MSI-H and TS** | | | | |
| Colorectal cancer | 13.86 (10.05 – 19.13) | <0.001 | 3,665 | No for benefit of combination of immunotherapy and fluorouracil/pemetrexed/capecitabine  (TS negativity is associated with response to fluorouracil/pemetrexed/capecitabine (9-12). However, data shows that TS positivity was correlated with TMB-H.) |
| Cholangiocarcinoma | 6.51 (1.25 – 34.07) | 0.022 | 400 |
| Epithelial ovarian Cancer | Infinity (>1.50)** | 0.031 | 632 |
| Female Genital Tract Malignancy | 1.74 (1.40 – 2.17) | <0.001 | 2,803 |
| Gastric cancer | 3.92 (1.91-8.06) | <0.001 | 483 |
| Neuroendocrine tumor | 4.33 (1.20-15.61) | 0.025 | 445 |
| Cancer with unknown primary | 10.36 (3.48-30.78) | <0.001 | 764 |
| Pancreatic cancer | 6.26 (2.30-16.99) | 0.001 | 1,189 |
| Small intestine cancer | 4.56 (1.67-12.43) | 0.0027 | 206 |
| **MSI-H and TOP2A** | | | | |
| Epithelial ovarian | 6.46 (1.56 – 26.73) | 0.002 | 3,751 | Yes for benefit of combination of immunotherapy and doxorubicin  (TOPO1 positivity, which is associated with doxorubicin response (7), was correlated with MSI-H.) |
| Female Genital Tract Malignancy | 1.82 (1.25 -2.64) | 0.001 | 2,817 |
| Gastric | 4.20 (0.99 – 17.83) | 0.043 | 480 |
| Neuroendocrine tumor | Infinity (>2.40)** | 0.004 | 441 |

*Fisher’s exact tests were performed for TS and TOP2A since tumor types could not be combined (only significant results are presented in table); if odds ratio of biomarker is less than 1 and p-value is significant, then biomarker negativity is associated with MSI-H

**Due to a zero cell, estimated odds ratio is infinite and the 95% confidence interval is one-sided

**Abbreviation**s: CI=confidence interval, MSI=microsatellite instability, TS=thymidylate synthetase; TOP2A=topoisomerase 2

**Supplemental Table 6: Relationship between TMB-H and protein markers***

|  | | **Odds Ratio**  **(95% CI)** | **p-value** | **N** | **Comment** |
| --- | --- | --- | --- | --- | --- |
| **TMB-H and RRM1 protein expression** | | | | | |
| Small intestinal cancer | 14.40 (2.33-89.03) | | 0.005 | 88 | No for benefit of combination of immunotherapy and gemcitabine.  (RRM1 negativity is associated with gemcitabine response (6). However, data shows that RRM1 positivity was correlated with TMB-H.) |
| Pancreatic cancer | 5.25 (1.90-14.49) | | 0.004 | 1,133 |
| Cancer with unknown primary | 1.93 (1.19-3.12) | | 0.010 | 717 |
| Non-small cell lung cancer | 1.34 (1.07-1.67) | | 0.012 | 4,072 |
| Female genital tract malignancy | 3.50 (2.76-4.44) | | <0.001 | 2,755 |
| Epithelial ovarian cancer | 3.77 (2.28-6.22) | | <0.001 | 3,664 |
| Breast cancer | 2.14 (1.03-4.45) | | 0.043 | 1,055 |
| Bladder cancer | 2.26 (1.18-4.32) | | 0.019 | 265 |
| Non-melanoma skin cancer | 0.38 (0.16-0.88) | | 0.031 | 105 | Yes for benefit of combination of immunotherapy and gemcitabine.  (RRM1 negativity is associated with gemcitabine response (6) and data shows that RRM1 negativity was correlated with TMB-H.) |
| **TMB-H and TS protein expression** | | | | | |
| Small Intestinal Cancer | 6.30 (1.97-20.10) | | 0.001 | 205 | No for benefit of combination of immunotherapy and fluorouracil/pemetrexed/capecitabine  (TS negativity is associated with response to fluorouracil/pemetrexed/capecitabine (9-12). However, data shows that TS positivity was correlated with TMB-H.) |
| Pancreatic cancer | 4.81 (1.89-12.27) | | 0.002 | 1,180 |
| Cancer with unknown primary | 2.08 (1.34-3.24) | | 0.001 | 755 |
| Non-small cell lung cancer | 1.63 (1.36-1.94) | | <0.001 | 4,231 |
| Gastric cancer | 4.47 (2.14-9.33) | | <0.001 | 481 |
| Female Genital Tract Malignancy | 1.68 (1.32-2.13) | | <0.001 | 2,791 |
| Epithelial ovarian cancer | Inf (>1.50)** | | 0.031 | 633 |
| Cholangiocarcinoma | 3.53 (1.20-10.42) | | 0.029 | 397 |
| Colorectal cancer | 10.57 (7.96-14.04) | | <0.001 | 3,652 |
| Breast cancer | 0.53 (0.30-0.95) | | 0.029 | 1,940 | Yes for benefit of combination of immunotherapy and fluorouracil/pemetrexed/capecitabine  (TS negativity is associated with response to fluorouracil/pemetrexed/capecitabine (9-12). Data shows that TS negativity was correlated with TMB-H.) |
| Merkel cell | 0 (<0.97)** | | 0.048 | 7 |
| **TMB-H and TUBB3 protein expression** | | | | | |
| Non-small cell lung cancer | | 1.22 (1.01-1.47) | 0.035 | 4,234 | No for benefit of combination of immunotherapy and taxanes  (TUBB3 positivity is associated with taxane resistance (13-15). Data shows that TUBB3 positivity was correlated with TMB-H.) |
| Melanoma | | 1.80 (1.30-2.48) | <0.001 | 802 |
| Female genital tract malignancy | | 0.78 (0.62-0.98) | 0.039 | 2,808 | Yes for benefit of combination of immunotherapy and taxanes  (TUBB3 positivity is associated with taxane resistance (13-15). Data shows that TUBB3 negativity was correlated with TMB-H.) |
| Colorectal cancer | | 0.43 (0.20-0.95) | 0.046 | 529 |

*Fisher’s exact tests were performed for RRM1, TS, and TUBB3 since tumor types could not be combined (only significant results are presented in table); if odds ratio of biomarker is greater than 1 and p-value is significant, then biomarker positivity is associated with TMB-H; if odds ratio of biomarker is less than 1 and p-value is significant, then biomarker negativity is associated with TMB-H

**Due to a zero cell, estimated odds ratio is zero and the 95% confidence interval is one-sided

**Abbreviations:** CI=confidence interval, RRM1=ribonucleotide reductase regulatory subunit M1, TMB=tumor mutational burden, TS=thymidylate synthetase; TUBB3=tubulin beta 3

**Supplemental Table 7**: Relationship between PD-L1 positivity and protein markers*

|  | | **Odds Ratio**  **(95% CI)** | **p-value** | **N** | **Comment** |
| --- | --- | --- | --- | --- | --- |
| **PD-L1 positive and ERCC1 protein expression** | | | | | |
| Glioblastoma multiforme | 1.61 (1.06 - 2.42) | | 0.030 | 900 | No for benefit of combination of immunotherapy and platinum  (ERCC1 negativity, which is associated with platinum response (1,2), however ERCC1 positivity was correlated with PD-L1 positivity.) |
| Female genital tract malignancy | 1.81 (1.31-2.49) | | <0.001 | 2,642 |  |
| Esophageal cancer | 2.07 (1.21-3.55) | | 0.010 | 486 |  |
| Gastrointestinal stromal tumor | 0.12 (0.014 - 0.99) | | 0.032 | 77 | Yes for benefit of combination of immunotherapy and platinum  (ERCC1 negativity, which is associated with platinum response (1,2), was correlated with PD-L1 positivity.) |
| **PD-L1 positive and RRM1 protein expression** | | | | | |
| Non-epithelial ovarian cancer | 13.64 (1.35-137.35) | | 0.025 | 126 | No benefit of combination of immunotherapy and gemcitabine.  (RRM1 negativity is associated with gemcitabine response (6); however data shows that RRM1 positivity was correlated with TMB-H.) |
| Soft tissue sarcoma | 2.34 (1.28-4.26) | | 0.010 | 537 |
| Pancreatic cancer | 1.84 (1.06 - 3.17) | | 0.032 | 1,114 |
| Female genital tract malignancy | 1.38 (1.07 -1.79) | | 0.016 | 2,710 |
| Cholangiocarcinoma | 5.06 (2.03 - 12.63) | | 0.002 | 372 |
| **PD-L1 positive and TOP2A protein expression** | | | | | |
| Non-epithelial ovarian cancer | Inf (>1.28)** | | 0.029 | 126 | Yes for benefit of combination of immunotherapy and doxorubicin  (TOPO2A positivity, which is associated with doxorubicin, epirubicin, and etoposide response (7,16), was correlated with PD-L1 positivity.) |
| Soft tissue sarcoma | 3.19 (1.71-5.96) | | <0.001 | 570 |
| Cancer with unknown primary | 17.76 (2.39-131.77) | | <0.001 | 253 |
| Non-melanoma skin | 3.78 (1.05-13.55) | | 0.045 | 140 |
| Non-small cell lung cancer | 8.55 (1.99-36.73) | | <0.001 | 214 |
| Neuroendocrine tumor | 5.53 (1.60 -19.16) | | 0.003 | 427 |
| Mesothelioma | 3.65 (1.34-9.94) | | 0.013 | 93 |
| Kidney cancer | 3.94 (2.26-6.87) | | <0.001 | 341 |
| Head and Neck | 3.61 (1.33 - 9.80) | | 0.010 | 191 |
| Female genital tract malignancy | 2.65 (1.58 - 4.45) | | <0.001 | 2,760 |
| **PD-L1 positive and TS protein expression** | | | | | |
| Small intestinal cancer | 3.47 (1.45-8.30) | | 0.007 | 205 | No for benefit of combination of immunotherapy and fluorouracil/pemetrexed/capecitabine  (TS negativity is associated with response to fluorouracil/pemetrexed/capecitabine (9-12). However, data shows that TS positivity was correlated with TMB-H.) |
| Pancreatic cancer | 2.40 (1.58-3.62) | | <0.001 | 1,163 |
| Cancer with unknown primary | 2.08 (1.46-2.97) | | <0.001 | 743 |
| Non-small cell lung cancer | 1.43 (1.03-2.00) | | 0.036 | 769 |
| Neuroendocrine tumor | 2.90 (1.17-7.15) | | 0.035 | 429 |
| Melanoma | 3.37 (1.35-8.43) | | 0.006 | 224 |
| Kidney cancer | 3.79 (1.39 - 10.32) | | 0.015 | 130 |
| Head and neck | 2.46 (1.68 - 3.61) | | <0.001 | 478 |
| Gastric cancer | 4.48 (2.34-8.55) | | <0.001 | 456 |
| Female genital tract malignancy | 1.32 (1.03-1.70) | | 0.031 | 2,747 |
| Epithelial ovarian cancer | 2.44 (1.25-4.77) | | 0.008 | 617 |
| Cholangiocarcinoma | 2.81 (1.40-5.62) | | 0.006 | 394 |
| Colorectal cancer | 3.50 (2.44-5.03) | | <0.001 | 3,566 |
| Breast cancer | 2.09 (1.44-3.04) | | <0.001 | 1,933 |
| Bladder cancer | 2.65 (1.53-4.59) | | <0.001 | 290 |
| **PD-L1 positive and TUBB3 protein expression** | | | | | |
| Soft tissue sarcoma | 1.79 (1.08-2.94) | | 0.027 | 568 | No for benefit of combination of immunotherapy and taxanes  (TUBB3 positivity is associated with taxane resistance (13-15). However, data shows that TUBB3 positivity was correlated with TMB-H.) |
| Cancer with unknown primary | 1.55 (1.07-2.24) | | 0.019 | 738 |
| Non-small cell lung cancer | 1.97 (1.36-2.84) | | <0.001 | 763 |
| Kidney cancer | 2.20 (1.26-3.84) | | 0.006 | 340 |
| Head and neck | 1.52 (1.04-2.21) | | 0.035 | 479 |
| Gastric cancer | 2.86 (1.55-5.27) | | 0.001 | 457 |
| Esophageal cancer | 2.23 (1.28-3.89) | | 0.004 | 490 |
| Bladder cancer | 2.50 (1.43-4.36) | | 0.001 | 291 |

*Fisher’s exact tests were performed for RRM1, TS, and TUBB3 since tumor types could not be combined (only significant results are presented in table); if odds ratio of biomarker is greater than 1 and p-value is significant, then biomarker positivity is associated with TMB-H; if odds ratio of biomarker is less than 1 and p-value is significant, then biomarker negativity is associated with TMB-H

**Due to a zero cell, estimated odds ratio is infinite and the 95% confidence interval is one-sided

**Abbreviations:** CI=confidence interval, ERCC1= excision repair complementation group 1, RRM1=ribonucleotide reductase regulatory subunit M1, TMB=tumor mutational burden, TS=thymidylate synthetase; TUBB3=tubulin beta 3
